# RAHI–SATHI Indo-U.S. Collaboration: The Evolution of a Trainee-Led Twinning Model in Global Health Into a Multidisciplinary Collaborative Program

**DOI:** 10.9745/GHSP-D-16-00190

**Published:** 2017-03-24

**Authors:** Apurv Soni, Nisha Fahey, Abraham Jaffe, Shyamsundar Raithatha, Nitin Raithatha, Anusha Prabhakaran, Tiffany A Moore Simas, Nancy Byatt, Jagdish Vankar, Michael Chin, Ajay G Phatak, Shirish Srivastava, David D McManus, Eileen O'Keefe, Harshil Patel, Niket Patel, Dharti Patel, Michaela Tracey, Jasmine A Khubchandani, Haley Newman, Allison Earon, Hannah Rosenfield, Anna Handorf, Brittany Novak, John Bostrom, Anindita Deb, Soaham Desai, Dipen Patel, Archana Nimbalkar, Kandarp Talati, Milagros Rosal, Patricia McQuilkin, Himanshu Pandya, Heena P Santry, Sunil Thanvi, Utpala Kharod, Melissa Fischer, Jeroan Allison, Somashekhar M Nimbalkar

**Affiliations:** a University of Massachusetts Medical School, Worcester, MA, USA.; b Des Moines University, Des Moines, IA, USA.; c Pramukhswami Medical College, Karamsad, India.; d Boston University, Boston, MA, USA.

## Abstract

**Background:**

In recent years there has been a surge in the number of global health programs operated by academic institutions. However, most of the existing programs describe partnerships that are primarily faculty-driven and supported by extramural funding.

**Program Description:**

Research and Advocacy for Health in India (RAHI, or “pathfinder” in Hindi) and Support and Action Towards Health-Equity in India (SATHI, or “partnership” in Hindi) are 2 interconnected, collaborative efforts between the University of Massachusetts Medical School (UMMS) and Charutar Arogya Mandal (CAM), a medical college and a tertiary care center in rural western India. The RAHI–SATHI program is the culmination of a series of student/trainee-led research and capacity strengthening initiatives that received institutional support in the form of faculty mentorship and seed funding. RAHI–SATHI's trainee-led twinning approach overcomes traditional barriers faced by global health programs. Trainees help mitigate geographical barriers by acting as a bridge between members from different institutions, garner cultural insight through their ability to immerse themselves in a community, and overcome expertise limitations through pre-planned structured mentorship from faculty of both institutions. Trainees play a central role in cultivating trust among the team members and, in the process, they acquire personal leadership skills that may benefit them in their future careers.

**Conclusion:**

This paradigm of trainee-led twinning partnership promotes sustainability in an uncertain funding climate and provides a roadmap for conducting foundational work that is essential for the development of a broad, university-wide global health program.

## Abstract (precis)

RAHI–SATHI presents an innovative twinning model of global health academic partnership, resulting in a number of successful research activities, that features trainees or students as the driving force, complemented by strategic institutional support from both sides of the partnership. Others can promote similar student-led initiatives by: (1) accepting an expanded role for trainees in global health programs, (2) creating structured research and program opportunities for trainees, (3) developing a network of faculty and trainees interested in global health, (4) sharing extramural global health funding opportunities with faculty and trainees, and (5) offering seed funding.

## BACKGROUND

Global health, described as a product of international and public health, has gained prominence over the past decade or so, specifically in academic centers.^1^ In the United States alone, there has been a tenfold increase in the number of global health programs from 2000 to 2012.^2^

Multiple factors underlie the surge in the number of global health programs in academic institutions, including an increased recognition of globally connected communities, a strong commitment to service and philanthropy, and growing student demand.^3^ These collaborative efforts have led to significant advances in understanding global health disparities as well as curbing epidemics of HIV/AIDS, malaria, tuberculosis, and, most recently, Ebola.^4^ Emerging evidence suggests that faculty and trainees worldwide also benefit from participating in these collaborations that transcend national borders^5^ by heightening their awareness of social determinants of health, increasing self-awareness, and broadening their perspectives.^6^

The current global health landscape represents a paradigm shift from the historical activities of international health.^7^ Specifically, the conventional model of professionals from high-income countries providing resources, services, and skills to “fix” problems ailing low- and middle-income countries are being replaced by a more bilateral, twinning approach whereby global and local entities share collective knowledge and resources to achieve a common goal.^8^^,^^9^ Twinning for global health, which has been used in the fields of emergency medicine, pediatric oncology, and medical education, emphasizes the role and value of the partner in the host setting where the majority of activities occur.^8^^–^^12^ However, most of the existing programs in peer-reviewed literature describe partnerships that are faculty-driven and supported by extramural sources of funding.^8^^–^^14^ Faculty-driven global health programs are often limited to the interests and expertise of the faculty, and thus a multidisciplinary approach becomes difficult.^7^

The conventional model of professionals from high-income countries “fixing” problems in low-income countries has been replaced by a twinning approach whereby global and local entities share collective knowledge and resources to achieve a common goal.

By contrast, we present a student/trainee-led Indo-U.S. initiative that organically followed the twinning model and gradually evolved into a multidisciplinary program. The program overcame resource limitations by positioning the trainees as the driving forces behind the collaboration, complemented by strategic institutional support and stewardship. To the best of our knowledge, our collaboration represents the first account of a trainee-led twinning program for global health. We believe this account can serve as a roadmap for other trainee-led initiatives to expand into larger institution-based global health programs with the help of faculty and institutional support.

We present a trainee-led Indo-U.S. twinning model that gradually evolved into a multidisciplinary program.

## THE RAHI–SATHI COLLABORATION

Research and Advocacy for Health in India (RAHI, which is the Hindi word for pathfinder) and Support and Action Towards Health-Equity in India (SATHI, the Hindi word for partnership) are 2 sister collaborations between the University of Massachusetts Medical School (UMMS) and Charutar Arogya Mandal (CAM), a charitable trust that operates a tertiary care center and medical school in rural western India. RAHI was formed in 2013 through a formal Memorandum of Understanding between UMMS and CAM to support research activities between the 2 institutions. RAHI currently focuses on maternal and child health and noncommunicable disease and injury through a number of research studies and programs. SATHI was formed in 2015 based on the experience and feedback from personnel involved in RAHI to support bilateral capacity strengthening activities, including trainee-exchange, structured mentorship, and biannual seminars on research and teaching methodology. The impetus behind SATHI was to train learners from diverse backgrounds to grow the collaboration. Educational leadership from UMMS and CAM formed a coalition to support this endeavor.

As the only publicly funded medical university in Massachusetts, UMMS strives to address health disparities locally in central Massachusetts and globally through its legacy partnerships with institutions from low- and middle-income countries. Similarly, CAM's mission is to care for the underserved of the community and train the next generation of health care providers for rural India. The Central Research Services and Community Extension Department are 2 specific examples of CAM's dedication for improving health of local Indian communities through community-centered research and service, respectively. Central Research Services was formed by CAM in 2009 in an effort to support investigator-initiated, community-based research studies and foster a research culture within the institution. The Community Extension Department is dedicated to delivering health programs to the local community. Through this department, CAM has established a network of Village Health Workers who receive training to perform disease screenings and deliver health education. Additionally, CAM operates 7 primary and secondary health centers within the region, which increases access to primary and specialist care among rural communities. The shared institutional commitment of UMMS and CAM to provide equitable health care to the community formed the bedrock of the RAHI–SATHI collaboration.

The RAHI–SATHI collaboration has been championed thus far by 2 trainee-leaders [A.S. and N.F.] who have led the overall RAHI–SATHI collaboration with a vision of trainee-centered research and outreach initiatives. These trainee-leaders began as undergraduate students at Boston University and transitioned to medical school at UMMS. The RAHI–SATHI collaboration also receives support from both predoctoral and postdoctoral trainees from UMMS and CAM who support specific ongoing projects or lead development of new research efforts.

The RAHI-SATHI collaboration is supported by both pre- and postdoctoral students from the United States and India.

The trainee-leaders recruited predoctoral and postdoctoral trainees by leveraging existing structured opportunities at UMMS and CAM. For example, UMMS medical students are required to participate in a scholarly endeavor as part of the longitudinal 4-year Capstone Scholarship and Discovery course. In addition, selected UMMS medical students in the Global Health Pathway are required to participate in a global health activity in the summer after their first year of medical school. Furthermore, UMMS surgical research scholars are required to dedicate 2 years of their residency program to conduct research in a full-time capacity. Finally, CAM residents are required to produce a scholarly dissertation at the end of their training. Through their participation, trainees contribute to research and public health activities that directly address the needs of the rural Indian communities while also meeting their own educational requirements and developing their personal research portfolio.

The trainees are supported by faculty at both UMMS and CAM (and initially at Boston University). The involvement of faculties from both institutions range from high-level advising on a specific project or scientific product to working closely in one-on-one mentorship capacity. Typically, faculty who assume a less-involved role, meet with the trainees once a semester to discuss overall progress and respond to questions and solicitations via email or teleconference. Meanwhile, faculty mentors meet with the students once a week or once every 2 weeks.

## TRAINEE-LED TWINNING MODEL FOR GLOBAL HEALTH

An international collaboration for strengthening emergency medicine in Ethiopia described 6 important phases of twinning^9^:

1. Initiate the partnership
2. Develop a shared work plan
3. Implement the program
4. Monitor outcomes
5. Evaluate results
6. Disseminate information

In this article, we describe the role of trainees as the driving force behind all 6 of these phases in the RAHI–SATHI twinning model for 4 different activities, selected specifically to illustrate the gradual evolution of the RAHI–SATHI collaboration and the transition of the workforce from the initial trainees to a new generation of trainees (Figure).

**FIGURE:**
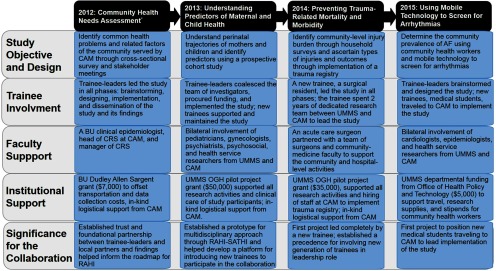
Timeline of RAHI–SATHI Evolution From Initial Study to a Multidisciplinary Collaboration Abbreviations: AF, atrial fibrillation; BU, Boston University; CAM, Charutar Arogya Mandal; CRS, Central Research Services; OGH, Office of Global Health; RAHI, Research and Advocacy for Health in India; SATHI, Support and Action Towards Health-Equity in India; UMMS, University of Massachusetts Medical School. *Study preceded RAHI–SATHI and was conducted by a joint collaboration between BU and CAM.

### Activity 1: Community Health Needs Assessment in Rural Western India: Cross-Sectional Survey of Women

**Phase 1: Initiate a partnership.** The official RAHI–SATHI collaboration was preceded by the foundational work of the 2 U.S. trainee-leaders [A.S. and N.F.], supported by their clinical epidemiology professor [E.O.] from Boston University, and 2 local leaders from the Central Research Services at CAM in India (the head [S.M.N.] and the manager [A.G.P.]). The 2 U.S. trainee-leaders were introduced to the Central Research Services leaders through a mutual acquaintance who was a faculty member at CAM. After discussions and identification of shared interests and local needs, the group decided to conduct an assessment of women's health status and determinants to produce a snapshot of the community health needs, which would also act as a roadmap for future collaborative activities.

**Phase 2: Develop a shared work plan.** The trainee-leaders developed a first draft of study materials based on feedback from U.S. and Indian investigators. The trainee-leaders' active role in consolidating feedback from Boston University and CAM faculty during this iterative process became a crucial learning experience in building consensus, anticipating real-world complications, and understanding the practical limitations of research. This approach also lessened the burden on the faculty, who were providing in-kind support to this study. The trainee-leaders also gained experience in grant writing while successfully applying for an institutional grant identified by their Boston University mentor.

**Phase 3: Implement the program.** The trainee-leaders were limited to 5 weeks of travel to India because of their employment and academic responsibilities. Therefore, the majority of the planning, including development of training protocols for research coordinators and a timeline of activities during the trainees' stay at CAM, were determined in the 3 months between submission of the research protocol to the Institutional Review Board (IRB) and approval. Aided by this preparation, the trainee-leaders interviewed, hired, and trained 5 female research coordinators in their first week onsite at CAM, and they supervised the research coordinators as they surveyed 700 reproductive-aged women from CAM outpatient clinics and surrounding villages over a period of 4 weeks.

**Phase 4: Monitor outcomes.** The trainee-leaders traveled with the research coordinators to the villages for community-based surveys. They spot-checked completed surveys in real-time and performed systematic quality checks on the surveys at the end of the day as well as during the data entry process. This exercise informed the trainees' understanding of research literacy among the research coordinators and participants. Their presence in the villages also provided the trainees an opportunity to interact with the local community and garner their thoughts on the concepts of health, public health, and research, which became instrumental in the development of subsequent projects.

**Phase 5: Evaluate results.** The trainee-leaders initially coordinated with statisticians from CAM and Boston University to derive findings from the data, led meetings with study investigators to discuss findings, and gathered feedback from the faculty to further guide the analysis. In the later stages, after cultivating analytic skills, the trainee-leaders assumed the role of performing analysis with the support of faculty-level analysts.

The cross-sectional survey identified a high burden of anemia, common mental disorders, chronic pain, and exposure to traumatic events. The factors participants identified as important for seeking health care were discordant with the focus of the Indian National Rural Health Mission. For instance, we found that mothers prioritized quality of care over other factors when considering care for their children, and they prioritized cost over other factors when seeking health care for themselves. By contrast, the government's policies for maternal and child health largely focus on reducing costs with limited strategies for improving the quality of care provided.^15^

This preliminary work identified 4 major needs, which serves as a roadmap for RAHI:

1. Understanding nutritional, psychosocial, and health care influences on mothers and children during the perinatal period in rural India.
2. Addressing mortality and long-term disability due to road-traffic accidents and other traumatic events.
3. Exploring the unique life-course and progression of cardiovascular and other noncommunicable diseases.
4. Identifying multidisciplinary approaches to combat chewable tobacco addiction among adolescents.

The preliminary work of the community health needs assessment identified 4 major needs, which serves as a roadmap for the RAHI collaboration.

**Phase 6: Disseminate information.** Findings were disseminated using 2 mechanisms to support broad communication and further develop the partnership:

1. Peer-reviewed publication of manuscripts and conference abstracts: The trainee-leaders developed first drafts of conference abstracts and manuscripts based on findings from the analysis and sought specific feedback from Boston University and CAM investigators and, at later stages, the UMMS faculty mentors.^15^^,^^16^
2. Discussion of findings with local stakeholders in rural India: The trainee-leaders returned to rural India for a period of 8 weeks to help disseminate the findings from the study to local stakeholders. Engaging officials in the public sector, including health care providers and government health officers, through email and telephone proved difficult and thus required in-person visits by the trainee-leaders and the head of Central Research Services who had a longstanding presence as a clinician in the community. Discussion topics were tailored to match the purview of the Chief District Health Officer, the Reproductive and Child Health Officer, and the District Development Officer. A semistructured interview format was abandoned in favor of a free-flowing conversation about their perception of community needs. The team presented findings from the cross-sectional survey in a large-group discussion format at CAM. The trainee-leaders met with clinicians from various clinical departments at CAM and local government primary care centers to ascertain their impressions about the clinical needs of their patient population and possible social determinants for health.

### Activity 2: Understanding Predictors of Maternal and Child Health in Rural Western India: Cohort Study of Pregnant Women

**Phase 1: Initiate a partnership.** In 2012, one of the trainee-leaders [A.S.] matriculated to UMMS, where he connected with the institutional Office of Global Health to identify resources and opportunities for collaboration. Together, both trainee-leaders [A.S. and N.F.] prepared a proposal to study predictors of maternal and child health in rural western India, the first need identified through their previous community assessment.

India bears the greatest burden worldwide of child malnourishment (about 52 million children have stunting) and mortality (in 2008, 1.8 million children under the age of 5 years died).^17^ Existing efforts made by the Indian government to improve health outcomes lack the support of evidence-based research.^18^ The underlying causes of poor maternal and child health and undernutrition may be multifactorial in nature, and an understanding of the experiences of Indian women throughout the perinatal period is necessary to help identify entry points for multifaceted interventions.^18^^,^^19^

In developing their proposal, the trainee-leaders worked with a faculty mentor [J.A.], who codirects the UMMS Center for Health Equity Intervention Research, and the head of Central Research Services at CAM [S.M.N]. Lead investigators of the study sought UMMS faculty support based on their expertise in peripartum health [T.M.S.], pediatrics [P.M.], women's mental health [N.B.], and psychosocial determinants of health [M.R.]. Based on their previous experience, the trainee-leaders and the head of Central Research Services at CAM actively recruited counterpart faculty from CAM [N.R., A.P., J.V.] to match UMMS faculty in content expertise, thereby facilitating an exchange of clinical and cultural knowledge. The research team successfully applied for seed funding through the UMMS Office of Global Health Pilot Project Grant to conduct a prospective cohort study of pregnant women in rural India.

**Phase 2: Develop a shared work plan.** Due to the broad scope of the study, the trainee-leaders met separately with faculty from each discipline to identify specific questionnaires, biomarkers, and measurements to be collected during the study. Trainee-leaders consolidated the feedback from the investigators and identified critical and optional components of the study. The trainee-leaders moderated group meetings among UMMS investigators and communicated with CAM investigators before and after group meetings to build consensus and finalize the type of information to be collected through the study. Thus, the trainee-leaders acted as a bridge between UMMS and CAM investigators as well as principal investigators and co-investigators. The success of trainee-leaders as mediators while developing a shared work plan among this multidisciplinary team collaborating on a project in rural India for the first time was predicated on 3 important factors: (1) building on preexisting relationships between the investigators at their respective institutions, (2) engaging faculty early in the process, and (3) demonstrating consistent progress on research design by the trainees at each meeting with faculty. Ultimately, we designed a prospective cohort study that recruited and followed pregnant women from the first trimester to 2 years postpartum with multiple data collection time points.

The trainee-leaders acted as a bridge between the 2 partner universities.

**Phase 3: Implement the program.** The trainee-leaders traveled to CAM during the summer after their first year of medical school to implement the study. The prospective cohort study required establishment of standard operating procedures, research space, and sustained administrative effort in India. The trainee-leaders and the CAM principal investigator held meetings with CAM leaders, administration, and faculty from relevant departments to describe the study and outline their roles for supporting the study. To assist with recruitment of pregnant women from the community, the research team, including trainee-leaders, coordinators, and the CAM principal investigator, organized a town hall meeting for community health workers of nearby villages to describe the study to them, their role of referring pregnant women to participate in the study, and the honorarium for supporting the study.

**Phase 4: Monitor outcomes.** The trainee-leaders developed standardized forms to track participant enrollment, follow-up visits, and clinical data, which were completed by research assistants on a weekly basis. However, long-term oversight by the trainee-leaders was difficult due to growing commitments from medical school as they transitioned from preclinical to clinical years. Therefore, they recruited a new generation of SATHI trainees to overtake monitoring of the study. Two graduate students from CAM [N.P. and H.P.] performed periodic data-entry checks and reviewed medical records of the participants. In addition, 4 UMMS medical students [M.T., H.N., J.K. and H.R.] traveled to CAM after their first year of medical school to support the research staff with data-entry and quality checks. In addition to monitoring outcomes of the study, the UMMS trainees also gained cultural and clinical insight into the research questions being investigated through the study. They used the experience to develop their research interests, which they will pursue for the remainder of their training at UMMS through their capstone course requirement.

**Phase 5: Evaluate results.** One of the trainee-leaders [A.S.] led the analysis of the emerging data by leveraging skills acquired through the doctoral program at UMMS Quantitative Health Sciences and with support from faculty-level statisticians. Additionally, the trainee-leader and his mentor [J.A.] conducted biannual workshops in statistical analysis at CAM to train local statisticians in intermediate and advanced analyses. The analytical team produced findings that were shared by the trainee-leaders with UMMS and CAM investigators, faculty, and students and continue to be disseminated through peer-review mechanisms.

**Phase 6: Disseminate information.** Early findings from the study have identified a high burden of low birth weight as well as low maternal hemoglobin and deficiency of essential vitamins during pregnancy despite the provision of prenatal care.^20^^–^^22^ Scholarly products, all first-authored by the trainee-leaders and second-generation trainees, were presented at scientific conferences, and 2 manuscripts are in development, which discuss the experiences of the pregnant women in rural western India.^20^^–^^22^ Trainees responsible for the first draft of the scholarly products solicited feedback from faculty at both institutions and incorporated their insights into the work. Additionally, preliminary data from this study played a vital role in successful applications by the CAM investigators [S.M.N., A.G.P, N.R., and A.P.] for research funding from the Indian Council of Medical Research to further study perinatal health.

### Activity 3: Reducing Injury-Related Morbidity and Mortality in Rural Western India: Community-Based Survey and Trauma Registry

**Phase 1: Initiate a partnership.** Prior to implementation of the maternal and child health study (activity 2), the trainee-leaders and the UMMS principal investigator hosted the CAM principal investigator for a seminar at the UMMS campus. The group presented findings of their community health needs assessment and outlined the scope of the new maternal and child health study. The seminar was attended by an acute care surgeon and health services researcher with roots in the CAM catchment area [H.P.S]. The surgeon expressed interest in supporting the collaboration. As director of Surgical Research Scholars, the surgeon recruited a surgical resident [A.J.] entering a 2-year period of dedicated research to lead a program addressing the trauma care needs in rural western India. This undertaking represents the first example of a new-generation trainee at the postdoctoral level leading the design and implementation of a project. The postdoctoral trainee traveled to CAM to gather local feedback about the knowledge gaps that could be addressed through the research study and identified faculty from the Department for Surgery [S.S.] and Community Extension Department [S.R.] to collaborate on the study.

**Phase 2: Develop a shared work plan.** During the postdoctoral trainee's visit to CAM, 2 major needs were identified: understanding injury burden at a community level and developing a mechanism to track and assess outcomes of trauma patients at CAM.

Road traffic accidents are a leading cause of mortality in India.^23^ One out of every 4 road traffic accidents in India results in a death, and nearly half of the fatal cases never receive any medical attention.^24^ CAM's hospital is situated at the intersection of 2 major roadways in rural western India. The hospital has more than 20,000 admissions per year, and an estimated 70% of these are attributed to road traffic accidents. However, the specific burden of trauma-related injuries and outcomes at the hospital and community level remain unknown. Trauma registries are an integral part of emergency care systems in high-income countries but not in low- and middle-income countries.^25^ They are important for quality improvement within an institution and surveillance of trauma-related outcomes.^26^ Therefore, the UMMS and CAM team decided to conduct a community-based survey and develop a hospital-based trauma registry, which was financially supported by RAHI–SATHI's second UMMS Office of Global Health Pilot Project Grant.

**Phase 3: Implement the program.** UMMS and CAM investigators leveraged a preexisting sampling frame created by CAM as part of their community outreach efforts to carry out burden of injury surveys among 5,000 household from 36 villages in the surrounding region. The postdoctoral trainee and CAM's Community Extension Department supervised this implementation. Diffusion of trauma registry, a new initiative within the busy setting of emergency care in a resource-limited setting, required buy-in at multiple levels, including the registrar, casualty medical officers, trauma specialists, and hospital leaders. The UMMS postdoctoral trainee dedicated 2 research years to systematically build buy-in for the trauma registry at CAM through a pilot implementation phase, discussions with physician champions, and stakeholder round-table forums. The principal investigator of the study [H.P.S], as director of the Surgical Research Scholars program, modified program requirements and sought support from UMMS leadership to accommodate the trainee's time at CAM. A first-year UMMS medical student [B.N], a member of the third-generation of predoctoral trainees, spent 4 weeks in India working with the postdoctoral trainee during the implementation phase. Ultimately, the trauma registry was implemented as standard operating procedures, replacing the existing intake form in the emergency department for all trauma-related injuries, thereby reducing the additional burden imposed on care providers and assuring maintenance by existing health care staff at CAM.

**Phases 4, 5, and 6: Monitor outcomes, evaluate results, and disseminate information.** The community health workers conducting surveys provided daily tallies of households surveyed, and weekly meetings were held to summarize the number of injuries and disabilities captured. Data are emerging from this study and have not yet been analyzed. However, the postdoctoral trainee and Department of Surgery at UMMS and CAM have shared their experience of studying trauma-related injuries in rural western India through departmental, institutional, and professional seminars. The understanding of barriers to trauma registry implementation identified in the pilot phase was shared at CAM as well as at meetings of the Massachusetts Committee on Trauma and the Association of Academic Surgery conference. Through this outreach, the UMMS team has identified the next surgical resident to assume responsibilities as the current postdoctoral trainee transitions back to clinical training.

### Activity 4: Detecting Unrecognized Atrial Fibrillation in Rural Western India Using Mobile-Based Technology: Community-Based Screening

**Phase 1: Initiate a partnership.** The trainee-leaders approached the chief of Connected Cardiovascular Healthcare section at UMMS [D.D.M.] to leverage his research team's expertise in mobile technology to collaborate with CAM for a joint Indo-U.S. call for proposals. Together, the group decided to focus on mobile-based screening of atrial fibrillation due to recent innovations at UMMS for this technology and its apparent need in India.

Atrial fibrillation is understudied among Indians but may be an underlying contributor to the ongoing stroke epidemic in India.^27^^,^^28^ Untreated atrial fibrillation can increase the risk of stroke, and in the context of India, where rapid stroke management is suboptimal, prevention of stroke through early diagnosis of atrial fibrillation becomes crucial.^29^ The technology developed by the UMMS atrial fibrillation research group in collaboration with local biomedical engineers uses a mobile phone to screen for atrial fibrillation.^30^ The trainee-leaders introduced the group to a member of the Office of Health and Technology at UMMS [M.C.] who codirects the Global Health Pathway at UMMS and thus oversees the SATHI component of the collaboration. The RAHI leadership team at CAM [S.M.N. and A.G.P.] recruited a cardiologist from CAM [S.T.] to collaborate on the study and provide clinical insight for conducting a feasibility study.

**Phase 2: Develop a shared work plan.** Conventionally, a 12-lead electrocardiogram (EKG) is required to obtain an electric signal of cardiac activity, which is interpreted by medically trained personnel to diagnose atrial fibrillation or other arrhythmias. However, a single screen for atrial fibrillation may miss cases of paroxysmal atrial fibrillation. Therefore, the research team decided to conduct a feasibility study that used algorithm-driven pulse waveform and single-lead EKG technology to screen participants for atrial fibrillation on 5 consecutive days. Partnership with CAM's Community Extension Department [S.R.] helped identify villages for community-based atrial fibrillation screening. CAM investigators suggested using community health workers to align the screening approach with the Indian government's model and develop a proof of concept that may be scalable across India.

**Phase 3: Implement the program.** Two first-year UMMS medical students [A.E. and A.H.], representing the third generation of SATHI trainees, were identified to implement the study. The trainees received training at UMMS by the study principal investigator, the trainee-leaders, and the atrial fibrillation research staff in using the mobile technology. They traveled to CAM with research equipment and materials with financial support from the UMMS Office of Health and Technology. They then trained research coordinators in the screening procedures. Ultimately, through a train-the-trainer model and medical students, local community health workers were able to recruit and screen more than 350 participants in their homes for 5 consecutive days. Although research design was formulated by the faculty principal investigator and the trainee-leaders, this pilot study represented the first account of a new generation of UMMS predoctoral trainees [A.E. and A.H.] leading the implementation of a study. It is noteworthy that neither of the trainees spoke the local Indian language. Partnering of UMMS and CAM trainees [D.P. and H.P.] helped overcome the linguistic and cultural implementation barriers.

**Phase 4, 5, and 6: Monitor outcomes, evaluate results, and disseminate information.** Data from this feasibility study was evaluated by a team of trainees with statistical [A.S.] and cardiovascular [N.F. and J.B.] training backgrounds. Early findings revealed a prevalence of atrial fibrillation substantially greater than previously reported in India and comparable with that found in the United States and other high-income countries. Although large-scale and more representative screening efforts are currently underway, noteworthy findings from the feasibility study were presented by a trainee-leader [A.S.] at the National Institutes of Health Special Topics Conference on Healthcare Innovations and Point-of-Care Technologies and were published in a leading mobile health journal.^31^ A grant proposal, coauthored by the trainee-leaders, to leverage public health infrastructure to establish a systematic screening program for atrial fibrillation was selected among the finalists for joint consideration by the National Institutes of Health and Indian Department of Biotechnology, but ultimately was not funded. The group is preparing a similar application in response to a call for proposals from the Fogarty International Center. Meanwhile, the UMMS Office of Global Health has awarded RAHI–SATHI its third Pilot Project Grant, which supports continued screening for atrial fibrillation using mobile technology based on the promising results from the feasibility study.

## SUCCESSES AND CHALLENGES OF A TRAINEE-LED TWINNING PROGRAM

Positioning trainees as the driving force behind a twinning program has its advantages. For example, the student trainees tend to have greater flexibility than faculty to travel to the local site, and they can facilitate an interdisciplinary approach by coordinating inputs from a range of faculty (Table 1). However, we also highlight 4 major challenges that trainee-led approaches must overcome:

1. **Time management:** Trainees experience time constraints as they seek to balance their formal training with participation in global health activities. Aligning trainees' responsibilities with their mandatory requirements for their formal training, such as capstone projects, can offer a possible solution for this challenge.
2. **Technical expertise:** Trainees have limited experience in conducting research, especially in a leading role. Pairing trainees with mentors and offering structured mentorship to support them in technical and team-building aspects can help overcome this knowledge gap.
3. **Identifying faculty mentors and champions:** Faculty members, especially physician scientists, experience time constraints with their clinical and research responsibilities, therefore limiting their capacity to accept mentees. Identifying grant and manuscript opportunities can propel faculty to be involved.
4. **Continuity:** Trainees' involvement in the project is confined to their time in the academic program. Presence of a faculty champion and explicit transition plans including supporting recruitment of subsequent generation of trainees can help maintain continuity.

**TABLE 1.** Advantages of a Trainee-Led Twinning Program in Global Health and Trainee Benefits Based on the RAHI–SATHI Experience

Twinning Phase	Advantages of Program	Benefits for Trainee
Initiate a partnership	Trainees typically have greater flexibility than faculty to travel and connect with the local community to build trust and act as a bridge between geographically separated investigators	Gain hands-on experience in professionalism and building trust with a new community
Develop a shared work plan	Trainees facilitate an interdisciplinary approach by coordinating inputs from multiple team members from varied disciplines and maximizing faculty investigators' contribution, in the context of their in-kind support	Acquire consensus-building skills and a multidisciplinary perspective
Implement the program	Trainees lead the implementation of the program and reduce the burden on international partners who face competing demands to provide care to their beneficiaries	Develop an understanding of real-world constraints and complications in implementation science
Monitor outcomes	Trainees identify potential sources of error early in the program and help sustain communication among the team and with the local community	Gain insight into potential pitfalls of programs
Evaluate results	Trainees provide insight gained by their involvement in the study and help guide evaluation of the results	Enhance scientific inquisition and analytical skills to interpret and discuss findings with peers
Disseminate information	Trainees disseminate information across a range of platforms including peer-reviewed manuscripts, conference presentations, and departmental or institutional seminars, resulting in greater exposure of the program and increased opportunities for potential collaborators to become involved	Develop scholarly skills that are critical in medicine and academics

Abbreviations: RAHI, Research and Advocacy for Health in India; SATHI, Support and Action Towards Health-Equity in India.

In addition to these 4 challenges, which are universal in nature, Indian trainees experienced unique challenges that limited their involvement. Medical education in India is intensely regulated by a national body, the Medical Council of India. Trainees' academic performance is heavily dependent on attendance and curricular-related activities, thereby limiting their elective time and participation in extracurricular scholarship. Despite our efforts to promote bilateral involvement, these constraints have limited more active participation of Indian trainees. However, in recent years there has been increased participation due to the combination of continued support from CAM's leadership and word-of-mouth promotion by CAM trainees who have participated in the RAHI–SATHI collaboration and accomplished peer-reviewed achievements in the form of published manuscripts and scientific conference presentations.

Presence of a faculty champion and explicit transition plans can help maintain continuity of trainee-led twinning models.

### Unsuccessful Proposals

Our collaboration prepared and submitted 4 unsuccessful proposals for extramural funding.

1. The “Biological Determinants of Type 2 Diabetes Risk in Indian Populations” proposal was developed in response to a joint Indo-U.S. call from the National Institutes of Health and the Indian Council of Medical Research. The proposal aimed to investigate differences in adipose tissue biology among non-Hispanic whites and South Asian Indians as a potential mechanism for high risk of diabetes among Indians with low body mass index (BMI).
2. The “Support and Action Towards Health-equity in India” proposal was developed in response to the Obama-Singh Initiative funding opportunity for higher-education programs. The capacity strengthening proposal preceded SATHI formation and included exchange and development program for trainees.
3. The “Strengthening Kangaroo Mother Care Implementation in Gujarat” proposal was developed in response to a solicited opportunity from the Bill & Melinda Gates Foundation. The proposal aimed to enhance hospital and community-based Kangaroo Mother Care.
4. The “***S***martphone ***M***onitoring for ***A***trial fibrillation in ***R***eal-***T***ime–**India** (**SMART**–**India**)” proposal was developed in response to a joint Indo-U.S. funding opportunity from the Indian Department of Biotechnology and the National Institutes of Health. This proposal sought to expand community-based screening of atrial fibrillation using mobile technology.

Despite these disappointing outcomes, the grant writing and submission process mobilized trainees and faculty members and ultimately provided an opportunity to overcome lack of funding by seeking alternate resources. The formation of SATHI despite the unsuccessful application provides a salient example of this approach. In addition to SATHI activities described above, a virtual development program in global health for trainees and faculty in neurology is underway through a bilateral partnership between junior faculty from UMMS and CAM [A.D. and S.D.]. Such contingency plans can help gather preliminary data and feasibility results, which is becoming increasingly important for extramural funding. Additionally, experience from other programs has suggested that continuous interaction and activities, independent of extramural support, builds and fortifies trust in an academic partnership.^32^

### Strategic Institutional Support

UMMS and CAM play a vital role in the growth and success of RAHI–SATHI by providing strategic support and fostering a collaborative environment that promotes faculty teamwork and encourages them to work with trainees. The Box outlines recommendations for the parent institutions and faculty mentors based on our experience with RAHI–SATHI.

BOX **Recommendations for Institutional Leadership and Faculty to Promote Establishment of Trainee-Led Twinning Programs for Global Health**

- Accept an expanded role for trainees in global health programs
- Create structured opportunities for trainees to engage in research and global health activities
- Develop a network of faculty and trainees interested in global health
- Share extramural global health funding opportunities with faculty and trainees
- Offer application-based opportunities to seed funding for global health activities and promote perseverance among partnerships that lack extramural support

Institutional leadership at both sites formalized the collaboration in its early stages through a memorandum of understanding, making it possible for the collaboration to harness existing institutional resources. Centers for Health-Equity Intervention Research (CHEIR) and Clinical and Translational Science (CCTS) have structured opportunities, which supported the trainees and faculty working with RAHI–SATHI. Additionally, the Office of Global Health at UMMS provided crucial seed funding to support the research studies. The International Medical Education Program partially supported trainees' travel to CAM. Similarly, CAM's leadership support of RAHI–SATHI positioned their faculty and trainees to assume an active and leading role in the collaboration. The establishment of Central Research Services in 2009 as part of the focus of the institution on development of research skills and projects among the faculty was germane to the support received by the research activities of RAHI–SATHI. CAM began funding internal research projects, capacity building in research and scientific writing, and also international travel to disseminate the work being done. The annual publication output by CAM in the last 3 years has more than quadrupled that in the past decade. The head of Central Research Services [S.M.N.] and his research team, including trainees, received complete financial support from CAM to travel to the United States for dissemination of their work at professional conferences such as the Pediatric Academic Societies and Consortium of Universities for Global Health. This travel fortified the interpersonal relationships between UMMS and CAM investigators and helped engage more faculty and trainees.

## CONCLUSION

This paradigm of trainee-led twinning partnership presents unique challenges and successes in addition to the ones experienced by traditional, faculty-led collaborative models. Trainees can help mitigate geographical barriers by acting as a bridge between members from different institutions, garner cultural insight through their ability to immerse themselves in a community, and overcome expertise limitations through pre-planned structured mentorship from faculty of both institutions. In the process, trainees can play a central role in cultivating trust among the team members and acquire personal leadership skills that may benefit them in their future careers. Our experience shows strategic institutional support to trainee-led initiatives in global health promotes sustainability in an uncertain funding climate and provides a roadmap for conducting foundational work that is essential for the development of a broad, university-wide global health program.

## Notes

**Peer Reviewed**
